# Amyand’s hernia: a 10-year experience with 6 cases

**DOI:** 10.1186/s12893-021-01306-z

**Published:** 2021-07-23

**Authors:** Yijie Gao, Taotao Zhang, Min Zhang, Zhengxu Hu, Qiao Li, Xiangwen Zhang

**Affiliations:** 1 Department of Gastroenterology Surgery, The Dalian Municipal Central Hospital Affiliated of Dalian Medical University, No. 826 Southwest Road Shahekou District, Dalian, 116033 People’s Republic of China; 2 Dalian Medical University, Dalian, 116044 Liaoning People’s Republic of China

**Keywords:** Amyand’s hernia, Appendectomy, Appendix, Hernia repair

## Abstract

**Purpose:**

Amyand’s hernia is a rare hernia defined as an inguinal hernia that contains the appendix within the hernia sac. Current treatment of Amyand’s hernia remains controversial. Our study retrospectively reviewed 6 cases of Amyand’s hernia, aiming to provide a reference for the surgical treatment of Amyand's hernia.

**Methods:**

Six patients diagnosed with Amyand’s hernia from September 2010 to May 2020 were retrospectively enrolled in our study. We summarized clinical data of six patients including the chief complaint, physical examinations, laboratory examinations, imaging examinations, surgical methods, and postoperative treatments and outcomes.

**Results:**

The diagnosis of six cases with Amyand’s hernia was made during surgery. Two patients had normal appendixes whereas the remaining four patients had appendicitis. Two patients with normal appendix received tension-free mesh repair through the inguinal incision. Among those with inflamed or perforated appendixes, two received mesh repair and the other two did not. The discharge time after surgery of six patients was 9.8 ± 6.1 days. One patient suffered from a wound infection. No additional postoperative complications were detected.

**Conclusions:**

Computed tomography and ultrasonography are helpful but limited in the definite diagnosis of Amyand’s hernia. The presence of a normal appendix does not require to be resected, but appendicectomy is necessary if the appendix is inflamed. The treatment of Amyand's hernia should be tailored based on the patient's condition and the type of Amyand's hernia.

## Introduction

Amyand’s hernia is defined as an inguinal hernia that contains the appendix within the hernia sac [1]. In 1735, C. Amyand first described an 11-year-old boy with an incarcerated inguinal hernia containing a perforated appendix [2]. Subsequently, this type of hernia was named Amyand’s hernia, which was rarely encountered in clinical practice. Amyand’s hernia occurs in only 1% (0.19–1.7%) of all inguinal hernia cases [3–6]. Moreover, Amyand’s hernia is classified into four subtypes regarding the clinical symptoms and the situation of the appendix (Table 1) [8]. There are no inflammatory changes in the groin of type 1 Amyand’s hernia; type 2 Amyand’s hernia is those in which the septic changes are confined to the hernia sac; type 3 Amyand’s hernia represents a scenario where the sepsis has spread beyond the hernia sac; type 4 Amyand’s hernia includes acute appendicitis and other abdominal lesions. The appendix within the hernia can be either normal or inflamed, in which 0.13% of cases have appendicitis. Notably, the perforation of the appendix could lead to a dramatic increase in the mortality rate (15–30%) due to severe abdominal sepsis [3–5, 7]. Commonly, the diagnosis of Amyand's hernia was made intraoperatively and few cases could be diagnosed before surgery. Besides, current management of Amyand's hernia remains controversial since different strategies should be tailored to different individuals. In this study, we reported six cases of Amyand's hernia and detailed different managements, aiming to provide a reference for the surgical treatment of Amyand's hernia.

**Table 1:** Four types of Amyand’s Hernia

Classification	Description	Surgical management
Type 1	Normal appendix with an inguinal hernia	Hernia reduction, mesh repair, appendectomy in young patients
Type 2	Acute appendicitis within an inguinal hernia, no abdominal sepsis	Appendectomy through hernia, primary endogenous repair of hernia, no mesh
Type 3	Acute appendicitis within an inguinal hernia, abdominal wall, or peritoneal sepsis	Laparotomy, appendectomy, primary repair of hernia, no mesh
Type 4	Acute appendicitis within an inguinal hernia, related or unrelated abdominal pathology	Manage as types 1 to 3 hernia, investigate or treat second pathology as appropriate

## Patients and methods

The study protocol, conform to the ethical guidelines of the 1975 Declaration of Helsinki, was approved by the Institutional Ethical Review Committee of The Dalian Municipal Central Hospital of Dalian Medical University. Written informed consents were obtained from all the participants. Six patients diagnosed with Amyand's hernia from September 2010 to May 2020 were retrospectively enrolled in our study. The following data were collected for further analysis: age, sex, chief complaint, clinical manifestations, white blood cell (WBC) and C-reactive protein (CRP) level at admission, diagnostic imaging, surgery, and postoperative outcome. The level of WBC and CRP of six patients were matched with the type of Amyand’s hernia which was diagnosed intraoperatively. WBC higher than 11 × 10^9^/L and CRP higher than 10 mg/L were defined as elevated, which indicated the existence of inflammation. The primary endpoint was 30-day hospital mortality. Secondary endpoints were postoperative surgical wound infection and hospital length after surgery.

## Results

### Characteristics of six patients

A total of six patients with Amyand’s hernia were admitted to the hospital. The patient with Case 3 were admitted to the hospital through the outpatient clinic, except for the other 5 patients who were admitted to the hospital in emergency department. All of them were males with the age of 64.0 ± 17.6 years old (range: 30–81 years old) (Table 2). One patient (16.7%) was admitted to the hospital because of right lower abdominal pain, and five patients (83.3%) were admitted because of a mass in their inguinal area and right lower abdominal pain.

**Table 2:** Baseline characteristics of six patients

Case No	Age (years old)	Sex	Clinical presentation	Diagnostic imaging	WBC and/or CRP	Amyand’s Hernia type
1	77	Male	Inguinal hernia associated with right lower quadrant abdominal pain	Abdominal CT	Elevated	2
2	64	Male	Inguinal hernia associated with right lower quadrant abdominal pain	Abdominal CT	Elevated	2
3	56	Male	Inguinal hernia associated with right lower quadrant abdominal pain	Ultrasonography	Normal	1
4	30	Male	Right lower abdominal pain	Ultrasonography	Elevated	2
5	77	Male	Inguinal hernia associated with right lower quadrant abdominal pain	Abdominal CT	Elevated	2
6	71	Male	Inguinal hernia associated with right lower quadrant abdominal pain	Abdominal CT	Normal	1

Appendix	Surgical approach	Appendicectomy	Herniorrhaphy technique	Drainage	Antibiotics after surgery	Surgical wound infection
Perforated	laparotomy	Yes	Bassini suture repair	Yes	Piperacillin sodium and sulbactam sodium combined with metronidazole	No
Perforated	laparotomy	Yes	Lichtenstein repair method	Yes	Piperacillin sodium and tazobactam sodium	No
Normal	laparotomy	No	Lichtenstein repair method	No	Cefazolin sodium pentahydrate	No
Inflamed	Laparoscopy	Yes	TAPP	Yes	Cefoperzone sodium and tazobactam sodium	No
Perforated	Laparoscopy	Yes	No repair	Yes	Cefoperzone sodium and tazobactam sodium	No
Normal	laparotomy	No	Lichtenstein repair method	Yes	Ceftriaxone combined with moxifloxacin	Yes

Discharge time after surgery (days)	Deaths					
15	No					
5	No					
3	No					
5	No					
11	No					
20	No					

### Preoperative examinations

**Fig. 1:**
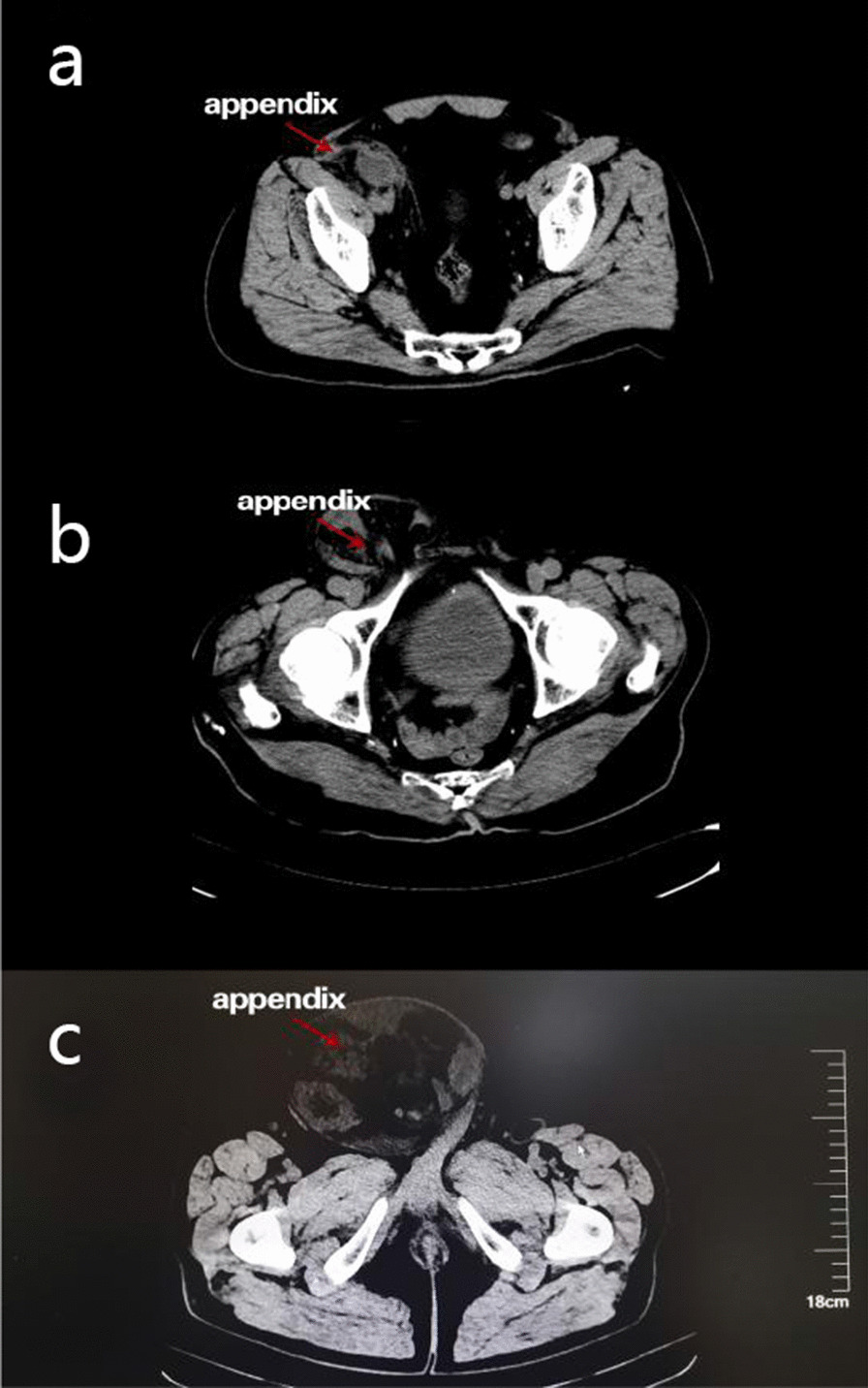
CT images of patients with Amyand’s hernia. **a** Preoperative CT images of appendicitis in Case 1. **b** Preoperative CT images indicated that the appendix was located in the hernia sac in Case 5. **c** Preoperative CT images indicated that the appendix was located in the hernia sac in Case 6

Six patients underwent computed tomography (CT) or ultrasonography examination before the operation. Four patients (Case 1, 2, 5, and 6) were diagnosed with appendicitis and inguinal hernia by abdominal CT. The appendix had a similar density compared with the surrounding hernia on CT images (Fig. 1). Case 3 was diagnosed with the inguinal hernia by ultrasonography whereas an enlarged appendix lumen and a right inguinal hernia were discovered by ultrasonography in Case 4. In laboratory examinations, WBC and CRP were helpful for distinguishing the inflamed appendix from the normal one. WBC and CRP were normal in two patients (Case 3 and 6) who had non-inflamed appendixes; in contrast, the other four patients (Case 1, 2, 4, and 5) with inflamed or perforated appendix exhibited elevated WBC and/or CRP.

### Operative methods

**Fig. 2:**
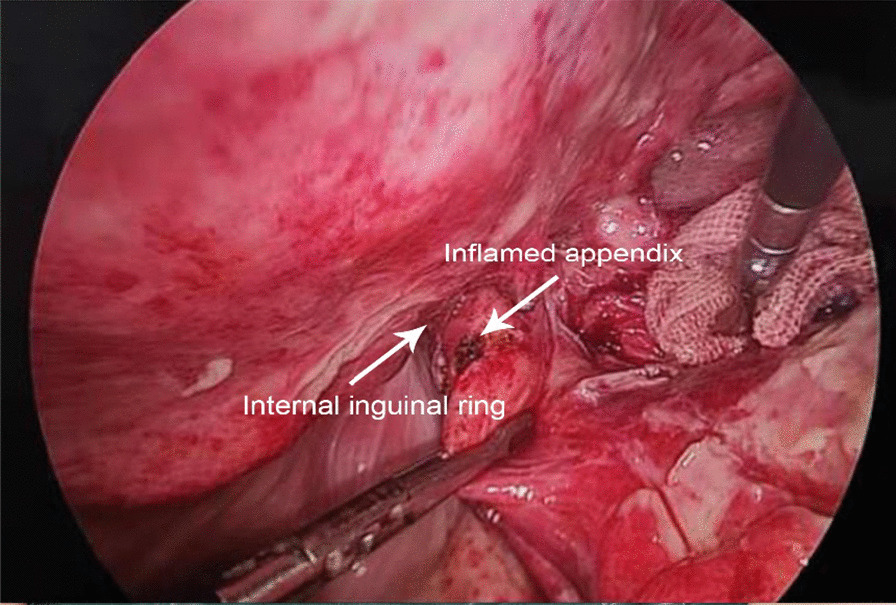
Appearance of inflamed appendix discovered during surgery. The inflamed appendix was separated from the hernia sac under the laparoscope in Case 5

Operative methods of six patients were outlined in Table 2. The diagnosis of Amyand's hernia of six patients was confirmed during surgery. Two patients whose laboratory examination was normal had normal appendixes, and appendicitis was detected in the remaining four patients (Fig. 2). Losanoff and Basson have described four subtypes of Amyand’s hernia and recommend different treatments (Table 1) [8]. Amyand’s hernia with a normal appendix is classified as type 1 whereas type 2–4 includes acute appendicitis. Therefore, Case 3, and 6 were classified as type 1 Amyand's hernia whereas Case 1, 2, 4, and 5 were type 2 Amyand's hernia. Among four patients with type 2 Amyand's hernia, the appendix of Case 1, 2, and 5 was perforated and that of Case 4 was only inflamed. In these cases, the surgical methods were determined according to the specific clinical features of the patients and the experience of the surgeon. When the patient had appendicitis, it was the priority to treat appendicitis and then deal with the hernia. Two patients (Case 3 and 6) who had normal appendixes received a Lichtenstein open repair method. However, those with inflamed or perforated appendix received different surgical procedures. Case 1 underwent appendicectomy and the Bassini suture open repair; Case 2 received appendicectomy and a Lichtenstein open repair method; Case 4 underwent laparoscopic appendicectomy and transabdominal preperitoneal prosthetic (TAPP); Case 5 received laparoscopic appendicectomy without further management of the hernia concerning the serious localized infection and inflammation. ProGripTM Self-Gripping Polypropylene Mesh was used for mesh repair. The drainage catheter was placed in the inguinal canal of most patients except Case 3.

### Postoperative outcomes

Postoperative management and outcomes were detailed in Table 2. The patients received different antibiotics after surgery. Case 6 suffered from incision infection after surgery whereas other patients did not. No other postoperative complications were detected in six patients. The discharge time after surgery of six patients was 9.8 ± 6.1 days (range: 3–20 days). Case 3 who had normal appendix had the shortest hospitalization time after surgery, whereas Case 6 who had normal appendix but developed incision infection had the longest hospitalization time after surgery. Patients who received inguinal incision had a longer median hospitalization time compared with those who received laparoscopy (10.8 ± 7.0 vs 8.0 ± 3.0 days). After discharge, all patients were followed up for more than one year through outpatient and telephone, and no recurrent hernia or delayed infection was found.

## Discussion

The pathogenesis of Amyand’s hernia associated acute appendicitis remains controversial. Previous studies indicated that muscle contractions or other sudden increases in intra-abdominal pressure might compress the appendix, resulting in inflammation [11, 12]. Moreover, an extraluminal obstruction of the appendix usually causes appendicitis due to pressure in the hernia neck rather than intraluminal obstruction [10, 11]. In our study, we presented six cases with Amyand’s hernia, in which two had normal appendix and four had appendicitis. The different operative methods and outcomes of six patients would provide a reference for the treatment of Amyand’s hernia.

A definitive preoperative diagnosis of Amyand's hernia is rare since the diagnosis is usually made during surgery. Physical examinations, laboratory examinations, and imaging examinations are not always associated with the differential diagnosis of Amyand's hernia. With respect to imaging examinations, CT scanning can facilitate the diagnosis of Amyand's hernia. However, CT is usually not the first choice for an uncomplicated inguinal hernia [13]. Therefore, the diagnosis of Amyand's hernia will be missed at that time. Sonography has been reported as a valuable examination in the preoperative screening of Amyand’s hernia since it is cheap and convenient for painful patients [14, 15]. Moreover, the suspected lesion can be further validated by CT. However, a preoperative diagnosis of Amyand's hernia based on ultrasound alone depends on the proficiency of the operator and, for that reason, remains a relatively unreliable imaging modality to accurately diagnose Amyand’s hernia [16]. Therefore, laparoscopic surgery can function as a diagnostic and therapeutic approach. Recently, a systematic review indicated that CT was the definitive diagnostic modality in patients with preoperative diagnosis [17]. In our six cases, four patients were diagnosed with appendicitis and inguinal hernia by CT whereas two patients were diagnosed with inguinal hernia by ultrasonography. For patients examined by CT, we all made the suspicion of Amyand’s hernia based on our experience but the definite diagnosis was made during the surgery since the density of appendix and small intestine was hard to differentiate by CT (Fig. 1). Therefore, we think that CT can facilitate the diagnosis of inguinal hernia but it is difficult to diagnose Amyand’s hernia before surgery. Among two patients received ultrasonography, one was diagnosed with Amyand’s hernia before surgery since the appendix was confirmed within the hernia. However, in another case, ultrasonography failed to detect the appendix and we did not suspect Amyand’s hernia before surgery. Therefore, we think that the diagnosis of Amyand’s hernia should be made by laparoscopy or laparotomy.

Generally, the primary management for Amyand’s hernia with a non-inflamed appendix is hernia repair without appendectomy [7, 18–20]. Some clinicians believe that this will decrease the occurrence of postoperative complications because appendectomy will convert a clean surgery into a clean-contaminated surgery. Also, the remaining appendix can be further used to replace the extrahepatic biliary tract, perform urinary diversion, or conduct Malone procedure [21, 22]. In our perspective, performing appendectomy in patients with previous inguinal hernia will increase the risk of hernia recurrence, since surgical manipulations at the base of the cecum may lead to deep inguinal ring detachment and subsequent inguinal hernia recurrence. Moreover, surgical manipulations involving the appendix might trigger secondary acute inflammation [22, 23]. However, these potential complications are minimized when the operation is performed laparoscopically [21, 22]. With the development of laparoscopic technology, laparoscopic appendectomy has become a surgical choice, especially in reducing surgical site infection, laparoscopic surgery has shown advantages [23]. In our experience of laparoscopic management of Amyand'shernia, for patients who need both appendectomy and hernia repair, we prefer to perform appendectomy first, disinfect the stump of the appendix, cover the stump of the appendix with purse suture, absorb the exudate from the abdominal cavity, and then repair the hernia. Of the six cases included in this study, the treatment of Case 3 and 6 was tension-free mesh repair without appendicectomy, which was consistent with the recommendation. As for other cases with type 2 Amyand’s hernia, Case 1 and 5 received appendicectomy without tension-free mesh repair, which was consistent with the recommendation. However, for Case 2 and 4 who had appendicitis, the surgeons performed appendectomy and tension-free mesh repair and patients did not develop postoperative infections. This may be due to postoperative antibiotics and pelvic drainage.

Prosthetic mesh is typically contraindicated in patients with an inflamed or perforated appendix because of the increased risk for wound and mesh infections [9]. Besides, a recent study suggested that mesh repair should be conducted after removal of the appendix regarding an inflamed appendix without perforation or abscess. As for the perforated appendix, the synthetic mesh repair should be avoided. Moreover, mesh repair should be deferred if the inguinal canal had severe inflammation [17]. In our four cases of mesh repair, polypropylene and polylactic acid composite mesh were used. In our view, the use of mesh repair should be tailored according to the physical condition of patients, the proficiency and experience of the surgeon, and the health care system of the hospital. Mesh repair is recommended when a non-inflamed appendix is discovered during herniorrhaphy. When acute appendicitis exists in the hernia sac, the surgeon should perform the appendicectomy and tension-free hernia repair. In our study, the appendix of Case 4 is inflamed but not perforated, therefore, the mesh repair is applicable in this case. Case 1, 2, and 5 have perforated appendix, in which Case 1 and 5 do not receive mesh repair whereas Case 2 receives mesh repair. Since Case 2 did not develop postoperative infections, the application of the drainage tube and antibiotics may be helpful for the prevention of infections. Besides, the surgeon did not perform hernia repair in Case 5 because of the serious infection of the inner ring. In this case, it is better to perform two-stage surgery or one-stage reopen hernia repair. However, additional studies are required to determine the optimal surgical approaches for these patients.

## Conclusions

Amyand's hernia is a rare presentation of inguinal hernias and the preoperative diagnosis of Amyand's hernia remains a challenge. CT and ultrasonography are helpful for the diagnosis but the definite diagnosis should be made by laparoscopy. The treatment of Amyand's hernia should be tailored based on the patient's condition and the type of Amyand's hernia. The application of tension-free mesh hernioplasty should be performed when the appendix is normally presented. If there is acute appendicitis in the hernia sac, appendectomy should be performed and the application of mesh repair should be carefully considered. Attention should be paid to the use of antibiotics and drainage in the operative area. We are still conservative about the application of mesh in hernia sac with acute appendicitis, which requires additional large-scale study to determine whether mesh repair will increase the risk of infection or not.

## Data Availability

All data generated or analysed during this study are included in this published article.
